# Dynamic Regulation of Grapevine’s microRNAs in Response to Mycorrhizal Symbiosis and High Temperature

**DOI:** 10.3390/plants12050982

**Published:** 2023-02-21

**Authors:** Catarina Campos, João Lucas Coito, Hélia Cardoso, Jorge Marques da Silva, Helena Sofia Pereira, Wanda Viegas, Amaia Nogales

**Affiliations:** 1 MED—Mediterranean Institute for Agriculture, Environment and Development & CHANGE—Global Change and Sustainability Institute, Institute for Advanced Studies and Research, Universidade de Évora, Pólo da Mitra, Ap. 94, 7006-554 Évora, Portugal; 2 LEAF—Linking Landscape, Environment, Agriculture and Food Research Center, Associate Laboratory TERRA, Instituto Superior de Agronomia, Universidade de Lisboa, Tapada da Ajuda, 1349-017 Lisboa, Portugal; 3 Department of Plant Biology/BioISI—Biosystems and Integrative Sciences Institute, Faculdade de Ciências, Universidade de Lisboa, 1749-016 Lisboa, Portugal

**Keywords:** *Vitis vinifera* L., microRNAs, *Rhizoglomus irregulare*, *Funneliformis mosseae*, heat-stress, stress response

## Abstract

MicroRNAs (miRNAs) are non-coding small RNAs that play crucial roles in plant development and stress responses and can regulate plant interactions with beneficial soil microorganisms such as arbuscular mycorrhizal fungi (AMF). To determine if root inoculation with distinct AMF species affected miRNA expression in grapevines subjected to high temperatures, RNA-seq was conducted in leaves of grapevines inoculated with either *Rhizoglomus irregulare* or *Funneliformis mosseae* and exposed to a high-temperature treatment (HTT) of 40 °C for 4 h per day for one week. Our results showed that mycorrhizal inoculation resulted in a better plant physiological response to HTT. Amongst the 195 identified miRNAs, 83 were considered isomiRs, suggesting that isomiRs can be biologically functional in plants. The number of differentially expressed miRNAs between temperatures was higher in mycorrhizal (28) than in non-inoculated plants (17). Several miR396 family members, which target homeobox-leucine zipper proteins, were only upregulated by HTT in mycorrhizal plants. Predicted targets of HTT-induced miRNAs in mycorrhizal plants queried to STRING DB formed networks for Cox complex, and growth and stress-related transcription factors such as SQUAMOSA promoter-binding-like-proteins, homeobox-leucine zipper proteins and auxin receptors. A further cluster related to DNA polymerase was found in *R. irregulare* inoculated plants. The results presented herein provide new insights into miRNA regulation in mycorrhizal grapevines under heat stress and can be the basis for functional studies of plant-AMF-stress interactions.

## 1. Introduction

Arbuscular mycorrhizal fungi (AMF) are soil-inhabiting fungi from the *Glomeromycota* phylum that form symbiotic associations with most terrestrial plants [1]. This relationship is based on the exchange of benefits between both partners: the plant supplies the fungus with carbohydrates derived from photosynthesis, and the fungus delivers mineral nutrients such as phosphorus (P) and nitrogen (N) to the plant [2,3]. Mycorrhizal symbioses are known to confer numerous advantages to plants, such as boosting photosynthesis, enhancing tolerance to various biotic and abiotic stresses, and improving soil-related aspects like soil aggregation, microbial diversity, and nutrient cycling [4,5,6,7,8,9,10].

The symbiotic association between plant and AMF is a highly regulated process that involves complex cellular reprogramming in both plant and fungal partners [11]. As previously described, hundreds of genes and metabolites are affected, starting at pre-infection stages and continuing through the various steps of the root colonization process [4,12,13]. After plant–mycorrhizae symbiosis is established, in aerial parts of the plants, there are changes in the expression of genes related to metabolic processes/pathways such as carbon assimilation and respiration, nutrient and water transport, hormonal metabolism, and stress response [14,15,16]. A strong molecular interaction must therefore exist between colonized roots and other plant tissues.

MicroRNAs (miRNAs) are single-stranded non-coding small RNAs, typically 21–24 nucleotide long, that down-regulate their target genes in plants mainly by transcript cleavage and less frequently through translation inhibition [17]. Evolutionary conserved miRNA families are found amongst plant species, sharing similarities at both sequence and target gene levels [18]. MicroRNAs have diverse roles in plants, from regulation of developmental processes [19,20] to physiological response/adaptation to environmental cues [21,22]. Importantly, miRNAs are known to be key regulators of plant biotic interactions, including those with beneficial microorganisms such as AMF [23,24,25]. In fact, it has been observed that miRNAs’ expression is highly altered in mycorrhizal plants when compared to non-mycorrhizal ones [23,25,26,27].

Understanding how plants sense and respond to abiotic stresses such as heat can have significant implications in plant breeding. In recent years, it has become clear that the roles of miRNA-guided gene regulation at transcriptional or post-transcriptional levels are critical for plant development and stress responses [28,29,30,31]. Several heat stress-regulated miRNAs have been identified, some of whose target genes are known to be involved in plant adaptation to high temperatures. For instance, upregulation of miR156 in *Arabidopsis* is related to heat stress tolerance and stress memory [32]. Other miRNAs such as miR159, miR166, miR393, miR396, and miR398, whose targets are involved in a variety of functions ranging from stress and disease tolerance to plant and floral development, have also been associated with heat stress [28,29,33].

As is the case for many other species, grapevine (*Vitis vinifera* L.) forms mycorrhizal symbioses which result in improved nutrition and water status for the plant, enhanced tolerance against pathogens (especially, root pathogenic fungi and nematodes), and abiotic stress response [34]. Amongst the latter, heat stress is a major cause for decreased growth, productivity, and grape berry quality in grapevine, since it negatively alters processes related to photosynthesis, cell redox state, respiration, and water-use efficiency of the plant [35,36]. Although mycorrhizal fungi are known to improve heat tolerance in grapevines as well as in other species, [37,38,39], the impact of mycorrhizae on plant miRNA regulation under heat stress is unknown. Moreover, molecular variability exists between distinct AMF species and even within different isolates of the same species [40], which can have different impacts on plant growth and physiological responses under abiotic stress conditions [41].

Considering these facts, in this study we hypothesized that there is considerable transcriptional variation of miRNAs in grapevine leaves related to mycorrhization and specifically to the different AMF species, which are most probably associated with a variable benefit to the plant. Hence, we aimed to evaluate (1) whether the inoculation with AMF induced changes in miRNA expression in grapevine leaves under long-term heat stress that could be associated with an increased physiological tolerance/adaptation to elevated temperatures, and (2) potential differences in miRNA expression and on plant response to heat stress induced by the inoculation of different AMF species—*Rhizoglomus irregulare* (Ri) or *Funneliformis mosseae* (Fm). The results obtained permit a better understanding of the molecular basis of mycorrhizal symbiosis, as well as of the mechanisms by which stress response/tolerance is improved in symbiotic plants. This, in turn, is essential for the development of new procedures in plant breeding programs directed toward enhancing plant tolerance to high temperatures.

## 2. Results

### 2.1. Mycorrhizal Inoculation Results in Better Plant Response to High Temperatures

Grapevines (var. Touriga Nacional grafted onto 1103 Paulse rootstock) were either inoculated with *R. irregulare* (Ri treatment) or *F. mosseae* (Fm treatment) and maintained in controlled conditions in a growth chamber for eight months. Non-inoculated plants were also grown as AMF-free controls. Subsequently, half of the plants were exposed to a high-temperature regime of 40 °C for 4 h per day for one week (HTT—40 °C), whereas the other half was kept at control temperature (CT—25 °C).

Grapevines inoculated with *R. irregulare* presented significantly higher root colonization rates than those inoculated with *F. mosseae* (*p* < 0.001): 0.83 ± 0.016 and 0.50 ± 0.065 for *Ri* and *Fm*, respectively. However, the one-week high temperature treatment did not have a significant effect on root colonization rates (*p* = 0.476), and there was not a significant interaction between mycorrhizal and temperature treatments. Non-inoculated plants did not present mycorrhizal colonization. The results of inoculation and heat stress treatment on plant physiology are shown in Table 1 and Figure S1.

Grapevine exposure to HTT—40 °C led to a decrease in water index (WI), normalized difference vegetation index (NDVI), and photochemical reflectance index (PRI), indicating that plants were at supra-optimal temperature conditions. The three reflectance indices have been used in previous works as estimates of plant stress status under different environmental conditions, including drought and heat stress [42,43,44]. No differences were observed among inoculated and non-inoculated plants.

Mycorrhizal inoculation positively affected net photosynthesis rate (P_n_) and chlorophyll index (CHLI) (Table 1, Figure S1). It is noteworthy that in plants exposed to high temperatures, an increase of 32% and 91% in P_n_ was observed in Ri and Fm plants, respectively, when compared to their own controls. This increase was less obvious in CHLI, which increased in 8 and 15% in Ri and Fm plants exposed to HTT—40 °C, respectively (Figure S1).

On the other hand, HTT—40 °C resulted in an increased grapevine transpiration rate (E), which was higher in Ri and Fm plants than in their respective controls (41 and 20% higher in Ri and Fm plants, respectively). Although the two-way ANOVA did not show a significant effect of HTT—40 °C or AMF factors on g_s_, under a high temperature, this parameter was 42% and 36% higher in Ri and Fm with respect to their controls.

### 2.2. Grapevine miRNA Profiling

Small RNA sequencing of the 24 grapevine libraries yielded between 9 to 14 million raw reads, with an average quality score Q30 of 98.2% (Table S2). After adaptor removal, quality control, and size filtering, a total of 195 miRNAs were obtained from the 24 libraries (Table S3), and the number of miRNAs amongst samples ranged from 106 to 132 (Table S2).

Length distribution analysis indicated that most miRNAs were in the 21 bp size class (Figure S2A), and 66.2% started with uridine, 16.2% with guanine, 9.6% with cytosine, and 8.1% adenine at the 5′-end (Figure S2B). MiRNAs belonged to 55 conserved miRNA families (Table S3), with the miR165/166 family being the most represented with 23 sequences, followed by miR156/157, miR159/319, and miR396 families, each with 16, 16, and 11 sequences, respectively (Table S3). However, there was great disparity in miRNA abundances within the same family, as exemplified by the counts for some miR319 members being amongst the lowest regarding all samples, along with members of the miR170/171, miR399, and miR156/157 families (Table S3).

Of the 195 miRNA sequences identified, 85 had a perfect match with grapevine mature sequences deposited in miRBase and were therefore annotated as canonical (Table S4). On the other hand, 83 sequences or length variants derived from a single locus (isomiRs) were identified (Table S3). Lastly, it was not possible to ascertain if the remaining 27 miRNAs sequences, which had high similarity to miRNAs identified in other plant species, corresponded to grapevine isomiRs, as there were no corresponding *V. vinifera* miRNA sequences deposited in miRBase. In general, the identified isomiRs differed among themselves by minor mismatches and indels. For example, all five sequences of miR319e (canonical and isomiRs, designated as 1 to 4) were derived from *locus* 11:4317298-4317320 (with minor mismatches) and all five sequences of miR159c-3p (canonical and isomiRs, designated 1 to 4) were derived from 17:2609214-2609236 (also, with minor mismatches) (Table S4). Members of the miR166 family were transcribed by 12 different miRNA coding loci and accounted for more than 82% of the total counts of miRNAs. In fact, for all treatments, the most abundant miRNA was the canonical miR166c-3p. This was followed by miR398c-3p except tor the Ri—40 °C treatment, where miR166h-3p.4 was the second most abundant (Table S3).

The expression of specific length variants appeared to be suppressed in some treatments. For instance, miR399 was only represented by the 21 nt class in CRi—40 °C samples, similarly to miR3627 in the CFm—40 °C treatment (Figure S3).

UpSetR analysis showed a high overlapping of miRNAs amongst all treatments (107) (Figure S4). This included miRNAs that regulate essential growth and developmental mechanisms in plants, such as miR156/157, miR159, miR166, miR167, miR168, and miR171, amongst others (Table S3). UpSetR analysis allowed for the determination of the number of miRNAs unique to each treatment, these being: seven for Cri—40 °C, five for Cri—25 °C and CFm—25°C, four for Ri—25 °C, and two for CFm—40 °C, Fm—25 °C and Fm—40 °C (Figure S4).

To identify possible novel miRNAs, the unclassified tags were further subjected to miRNA prediction using stringent criteria [45]. To enhance predictive accuracy in the identification of novel miRNAs, the miRNA/miRNA* criterion was the primary consideration in our analysis. We predicted 32 novel miRNAs (Table S5), of which 6 were found to be already deposited in the miRVIT database [46]. Of the 32 novel miRNAs, 17 were only found in one sample, and at low levels (Table S5). Several novel miRNAs were derived from the same locus (with minor mismatches) and may represent different isomiRs (Vvi-7, Vvi-8, and Vvi-17 for one locus and Vvi-14 and Vvi-26 for the other locus) (Table S5).

### 2.3. Heat- and AMF-Responsive miRNAs in Grapevine Leaves

**Figure 1:**
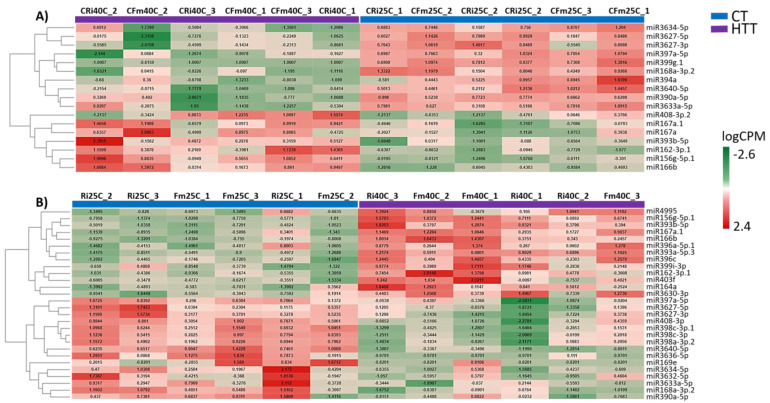
Heatmap showing differentially expressed miRNAs in (**A**) non–mycorrhizal and (**B**) mycorrhizal grapevines, maintained at control temperature (CT—25 °C) or subjected to a high–temperature treatment (HTT—40 °C).

To better understand if the temperature and AMF inoculation treatments had an influence on miRNA expression in grapevine leaves, we compared their expression levels among the different treatments through GLM Likelihood Ratio test. The direct comparison between mycorrhizal vs non-mycorrhizal plants in control temperature revealed that miR399g.1 and miR396c were downregulated by inoculation (FDR < 0.05). We then looked at the effect of high temperature in either non-mycorrhizal or mycorrhizal plants (Figure 1). This analysis showed a higher number of differentially expressed microRNAs (DEmiRNAs) in mycorrhizal (28) than in non-mycorrhizal plants (17) exposed to heat (Figure 1, Tables S6 and S7). In non-mycorrhizal plants exposed to HTT—40 °C, the most expressed miRNAs included miR408-3p.3, miR167a, and miR167a.1, miR393b-5p, miR162-3p.1, miR156g-5p.1, and miR166b (FDR < 0.05) (Figure 1A). MiRNAs induced by HTT—40 °C in mycorrhizal plants further included miR164a, miR3630-3p, miR396 family members, miR399i-3p, miR393a-5p.3, miR403f, and miR4995 (Figure 1B). On the other hand, miR3627 (-5p and -3p), miR3640-5p, miR3634-5p, and miR397a-5p were consistently downregulated by heat in both mycorrhizal inoculation treatments. Moreover, several members of the miR398 family were found to be downregulated by HTT—40 °C only in AMF inoculated plants (miR398c-3p, miR398c-3p.1, and miR398a-3p.2) (Figure 1B).

**Figure 2:**
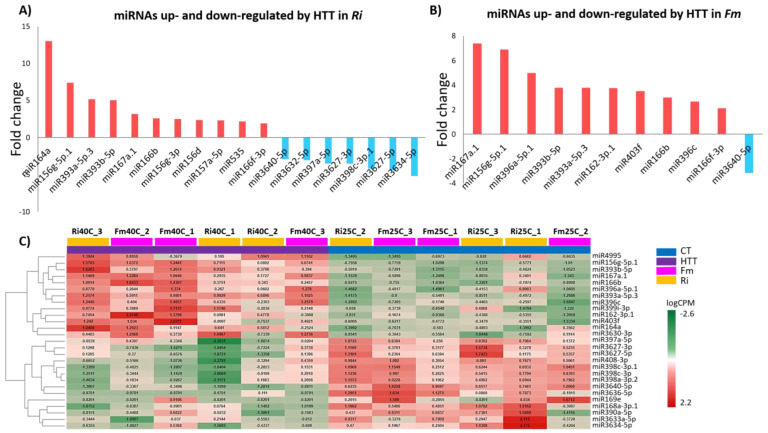
MiRNAs up- or downregulated by high-temperature treatment (HTT—40 °C) in mycorrhizal plants: (**A**) miRNAs up- and downregulated by HTT—40 °C in *R. irregulare* inoculated plants, (**B**) miRNAs up- and downregulated by HTT—40 °C in *F. mosseae* inoculated plants, and (**C**) Heatmap of GLM analysis showing differentially expressed miRNAs in all mycorrhizal plants, maintained at control temperature (CT—25 °C) or subjected to HTT—40 °C.

We then investigated if there were differences in heat-modulated miRNAs between Fm and Ri plants. EDGE analysis found 18 DEmiRNAs (FDR < 0.05) between control temperature and HTT- 40°C treatments in Ri plants and 11 in the Fm plants. The miRNAs upregulated by HTT- 40°C with highest fold-change were miR164a and miR167a.1 for Ri and Fm plants, respectively. Interestingly, several miRNAs belonging to the miR156/miR529/miR535 superfamily, which is known to be involved in plant stress responses [32], were found to be upregulated by heat stress in Ri plants (miR156d and miR535). MiR3640-5p was the only miRNA downregulated by heat stress in Fm, but in Ri several others were also affected: miR3632-5p, miR397a-5p, miR3627-3p, miR398c-3p.1, miR3627-5p, and miR3634-5p (Figure 2A,B).

GLM analysis further showed the interaction of temperature and mycorrhizal treatments on miRNA expression (Figure 2C, Table S8). The heatmap in Figure 2C shows, for instance, that miR408-3p, miR390a-5p, and miR3633a-5p responded to both temperature and mycorrhizal treatment (FDR < 0.05). Canonical miR398c-3p and isomiR miR398c-3p.1 were downregulated by heat stress.

The predicted novel miRNA Vvi-4 was greatly upregulated by HTT—40 °C (FDR < 0.05), regardless of whether plants were inoculated or not with AMF (Table S5).

### 2.4. Prediction of Grapevine miRNA Target Genes

The results of psRNATarget analysis for target gene prediction of grapevine miRNAs are shown in Table S9, and are in agreement with previous studies [47,48]. Amongst the miRNAs upregulated by high-temperature treatment, both in non-mycorrhizal and mycorrhizal plants, miR166b was predicted to target several genes encoding for homeobox-leucine zipper proteins (ATHB-15, REVOLUTA, HOX-32) and a TMV resistance protein N, and mir167a.1 was predicted to target signal peptidase complex subunit 3B (Table S9). In contrast, miR3627-5p and -3p, which were repressed by heat both in non-mycorrhizal and mycorrhizal plants, were predicted to target genes encoding wall-associated receptor kinase-like 8 and tyrosine-protein phosphatase, respectively. MiR3634-5p, which was also consistently downregulated by HTT—40 °C, was predicted to target genes encoding E3 ubiquitin-protein ligase CHFR, eukaryotic translation initiation factor, and protein argonaut 5 (Table S9). MiR397a-5p was predicted to target several *laccase* genes and two-component response regulator ARR22. MiR3640-5p, which was also downregulated by HTT—40 °C in both treatments, was predicted to target a gene encoding thioredoxin-like protein slr0233, the transcription factor bHLH128, and kinesin-like protein KIN-UB, amongst others. Members of the miR398 family, which were only repressed by HTT—40 °C in AMF-inoculated plants, were predicted to target genes encoding cytochrome c oxidase (Cox) and serine/threonine-protein kinase PBS1 (Table S9). Temperature also increased miRNAs from the miR399 family, which were predicted to target an inorganic phosphate transporter 1-3-like (Table S9). MiR3633a-5p, which was repressed by temperature, was predicted to target gibberellin 2-beta-dioxygenase, as previously observed [47].

MiR164a, only induced by heat in the *R. irregulare*-inoculated plants, was predicted to target genes encoding nascent polypeptide-associated complex (NAC) proteins, CUP-SHAPED COTYLEDON 2, Growth-Regulating Factor 8 (GRF8), and GDSL esterase/lipase (Table S9). MiR396 members upregulated in Fm plants were predicted to target genes encoding G patch domain-containing protein (TGH), bZIP transcription factor 16, a disease resistance protein, and GRF family members (Table S9). Members of the miR156/157 family were predicted to target genes encoding SQUAMOSA-promoter binding like-protein (SPL), as well as abscisic acid 8’-hydroxylase 4 and importin subunit beta-1 (Table S9). MiR393a/b, upregulated by HTT—40 °C in both treatments, were predicted to target genes for protein Transport Inhibitor Response 1 (TIR1), protein auxin signalling F-BOX 2 (AFB2), and Cox subunit 6a. MiR3632-5p, downregulated only in Ri plants, was predicted to target proteasome subunit alpha type-1-B-like and rRNA-processing protein FCF1 homolog (Table S9).

The novel miRNA Vvi-4 was predicted to target a putative disease resistance RPP13-like protein 1 (Table S5).

**Figure 3:**
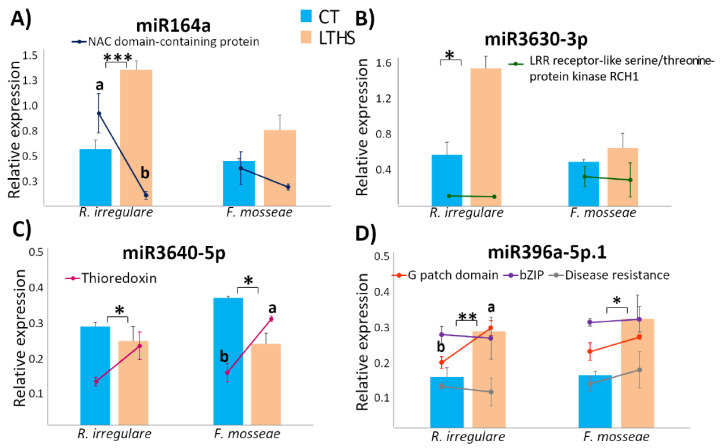
Expression of grapevine miRNAs: (**A**) miR164a, (**B**), miR3630-3p, (**C**), miR3640-5p and (**D**) miR396a-5p.1., and respective target genes, from plants maintained at control temperature (CT—25 °C) or subjected to high–temperature treatment (HTT—40 °C). Significant differences in miRNA expression between mycorrhizal/non–mycorrhizal or heat–treated/non–heat treated plants are indicated by * (*p* < 0.05), ** (*p* < 0.01) or *** (*p* < 0.001). Different letters denote significant differences in target gene expression levels (*p* < 0.05). Different letters indicate significant differences at *p* < 0.05.

The expression of four miRNAs and their potential target genes was further validated by RT-qPCR. Expression levels of miR164a, miR3630-3p, miR3640-5p, and miR396a-5p.1 were consistent with the miRNA-seq results (Figure 3). The expression of targets genes of miR164a, miR3630-3p, and miR3640-5p (encoding LRR receptor-like serine/threonine-protein kinase RCH1, NAC domain-containing protein 100, and thioredoxin-like protein slr0233, respectively) showed a negative correlation with the corresponding miRNA (Figure 3A–C). Three targets for miR396a-5p (G patch domain-containing protein TGH, bZIP transcription factor 16, and disease resistance protein) showed variable expression patterns amongst Ri and Fm plants (Figure 3D).

### 2.5. GO and KEGG Functional Analyses of miRNA Target Genes

**Figure 4:**
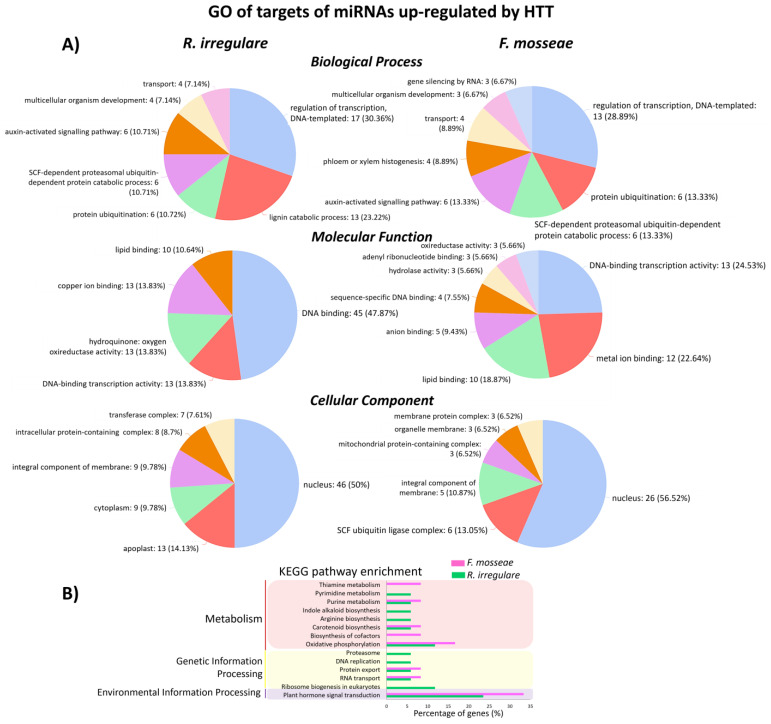
(**A**) Pie-charts demonstrating GO enrichment analysis and (**B**) KEGG pathway enrichment for target genes of miRNAs upregulated by HTT—40 °C in *R. irregulare*- and *F. mosseae*-inoculated grapevine plants. GO terms are shown sorted highest to lowest.

**Figure 5:**
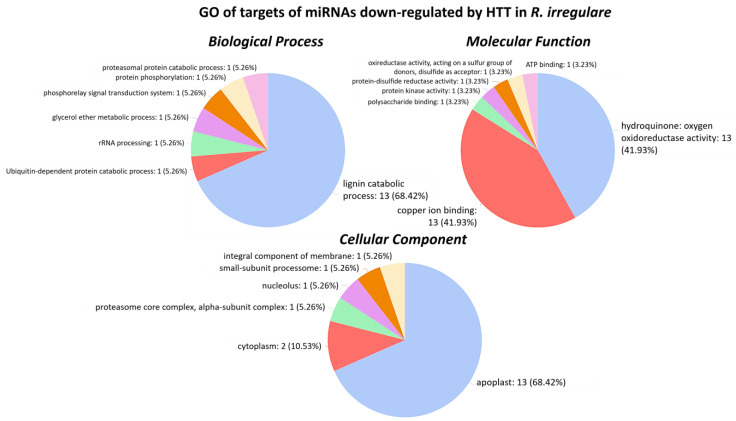
Pie charts of GO enrichment for target genes of miRNAs downregulated by HTT—40 °C in *R. irregulare*-inoculated grapevine plants. GO terms are shown sorted highest to lowest.

To have an overview of the functions of target genes of conserved miRNAs, we further performed a Gene Ontology (GO) enrichment analysis (Figure 4, Figure 5 and Figure S5). Differences in GO terms were found for the targets of HTT—40 °C upregulated miRNAs, between Ri and Fm plants (Figure 4A). In the Biological Process class, Fm treatment was enriched in ‘phloem or xylem histogenesis’ and ‘gene silencing by RNA’, whereas Ri was not. In the Molecular Function class, plants inoculated with *R. irregulare* were highly enriched in ‘DNA binding’, and *F. mosseae*-inoculated plants had enriched terms such as ‘hydrolase activity’, ‘adenyl ribonucleotide binding’, and ‘oxireductase activity’. In the Cellular Component class, ‘nucleus’ was the most enriched term in both treatments, followed by ‘apoplast’ and ‘SCF ubiquitin ligase complex’ in Ri and Fm plants, respectively (Figure 4A). The terms ‘cytoplasm’, ‘transferase complex’, and ‘intracellular protein-containing complex’ were unique to *Ri*, whereas ‘mitochondrial protein-containing complex’, ‘organelle membrane’ and ‘membrane protein complex’ were enriched in Fm.

Kyoto Encyclopaedia of Genes and Genomes (KEGG) pathway enrichment analysis showed that target genes of the HTT-upregulated miRNAs participated in several processes from Metabolism, Genetic Information Processing, and Environmental Information Processing pathways (Figure 4B). The terms ‘pyrimidine metabolism’, ‘indole alkaloid biosynthesis’, ‘arginine biosynthesis’, ‘proteasome’, ‘DNA replication’, and ‘ribosome biogenesis in eukaryotes’ terms were exclusively enriched in Ri plants, whereas ‘thiamine metabolism’ and ‘biosynthesis of cofactors’ were exclusively enriched in Fm plants (Figure 4B).

The GO terms relative to the targets of HTT-downregulated miRNAs in *R. irregulare* inoculated plants are shown in Figure 5. In the Biological Process class, the term ‘lignin catabolic process’ was highly enriched, and other catabolic terms were also found. In the Molecular Function class, ‘hydroquinone: oxygen oxidoreductase activity’ and ‘copper ion binding’ comprised most of the sequences, and ‘apoplast’ was the most enriched term in the Cellular Component class, followed by ‘cytoplasm’. In contrast, in Fm plants, only miR3640-5p was found to be significantly repressed by HTT—40 °C (Figure S5). Terms like ‘root development’ and processes related to microtubule were the most enriched in these plants.

### 2.6. Prediction of Heat-Associated Regulatory Networks

**Figure 6:**
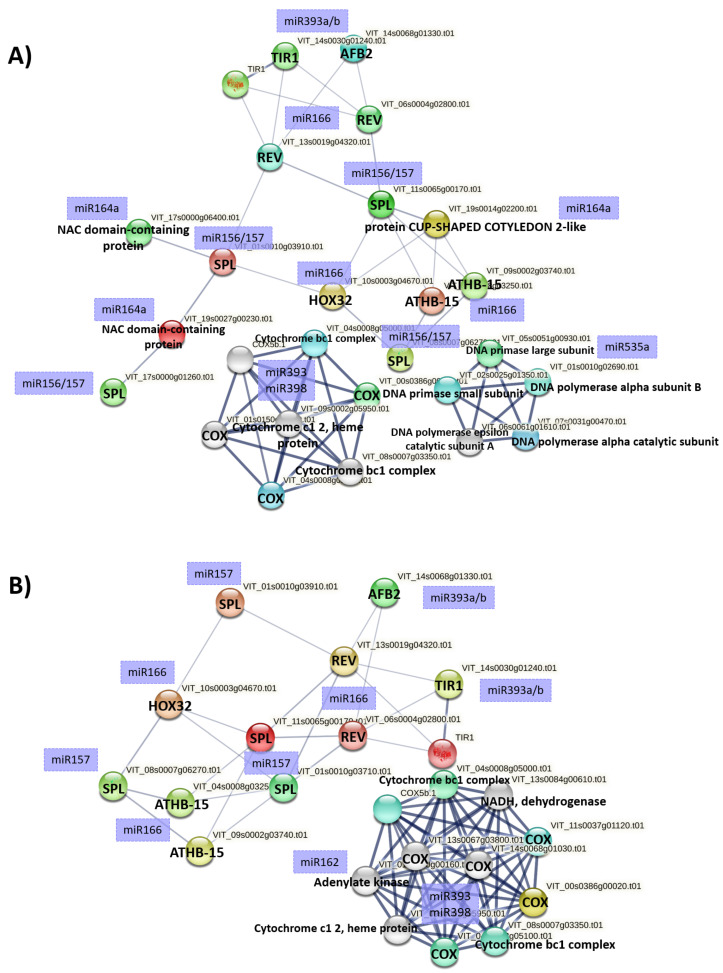
STRING network view of known and predicted interaction partners for targets of miRNAs upregulated by HTT—40 °C in (**A**) *R. irregulare,* and (**B**) *F. mosseae* inoculated plants. Predicted miRNA targets were utilized to query orthologous proteins in grapevine. The confidence view screen shows established and predicted protein–protein interactions, with stronger associations represented by thicker lines (edge confidence 0.900).

Predicted targets of miRNAs upregulated by HTT—40 °C in Ri and Fm plants queried to STRING DB predominantly formed networks for the Cox complex, a major site for oxidative phosphorylation, and growth and stress-related transcription factors such as SQUAMOSA Promoter-Binding-Like-Proteins, Homeobox-leucine zipper proteins (ATHB-15, REVOLUTA, HOX32), and auxin receptors such as auxin signalling factor F-box 2 and TIR1 (Figure 6A,B). A further interaction cluster related to DNA polymerase was also detected in plants inoculated with *R. irregulare* (Figure 6A).

## 3. Discussion

### 3.1. Mycorrhizal Plants Show Enhanced Response to High Temperature

Reflectance indices are a fast and non-destructive way to assess plant stress status under different environmental conditions, including drought and heat stress [42,43,44]. In the present experiment, the values of WI, NDVI, and PRI indices were significantly affected by the applied HTT—40 °C, and therefore, they indicated that plants were at supra-optimal temperature conditions.

However, gas exchange parameters such as P_n_ or g_s_ were not significantly affected by the temperature treatment, and E responded positively to it. The increase in E is the passive result of an increased vapor pressure deficit between the leaf and the atmosphere due to higher ambient temperature, but it could have an increased leaf evaporative cooling [39,49]. Increased transpiration might have slightly decreased the relative water content of leaves, as indicated by lowered WI. The decrease in NDVI and CI indicates a decrease in leaf chlorophyll content, and the decrease in PRI arguably indicates a decrease in light use efficiency, showing that at HTT—40 °C plants were under thermal stress. However, since the leaf chlorophyll content is frequently in excess of the needs of the photosynthetic activity, and light use efficiency is detected only under very low photosynthetic active radiation, these decreases were not translated to the measured leaf photosynthesis.

Similarly to other studies [37,38,39,50], in the present work, we observed that grapevine inoculation with AMF (both for *R. irregulare* and *F. mosseae*) increased plant ability to adapt to moderately high temperatures, as demonstrated by their higher P_n_, g_s_, and E in comparison to non-inoculated plants under the HTT—40 °C (Figure S1). Moreover, we found a higher CHLI index in mycorrhizal plants, suggesting that AMF-inoculated plants were able to avoid the reduction in chlorophyll content commonly observed under heat stress conditions [51,52].

### 3.2. Heat Stress Differently Affects miRNA Regulation in Mycorrhizal vs. Non-Mycorrhizal Grapevines

In this work, we identified 195 conserved miRNAs and 33 putative novel miRNAs in grapevine leaves (Tables S4 and S5). Interestingly, there were differences in the abundance of known miRNAs of the same family amongst experimental treatments, which may be explained by the fact that miRNA genes rely on their own promoter activity for transcription regulation [53]. MicroRNA genes are mostly transcribed from independent non-protein-coding loci, present in intergenic genomic regions [54]. In the case of isomiRs, their biogenesis is thought to be due to variations in the cleavage of pre-miRNA into a mature miRNA duplex by DICER-LIKE 1 [55]. The significance of isomiRs in plants is still under discussion; however, the fact that they are loaded into AGO proteins in RISC and present differential mRNA targeting and expression patterns strongly suggests that isomiRs are biologically functional and important in plants [55]. It has also been shown that isomiRs’ accumulation varies in response to temperature stress [30,56], indicating the need for quantifying isomiRs in an integrated analysis of miRNAs’ functions. The high number of isomiRs that we identified in the 24 grapevine libraries (83 out of 195 miRNA sequences) is in the range of what has been previously observed in wheat [30]. In *Pinus pinaster*, a large number of isomiRs were found to respond to drought, where the accumulation of isomiRs was related to higher or lower drought tolerance in different pine genotypes [57]. It has been suggested that isomiRs can extend the canonical miRNA regulatory network and that a single precursor sequence can be cleaved into more than one biologically meaningful product [58]. IsomiRs were also found to respond to drought stress in rice, and target genes were identified for several isomiRs, confirming their biological activity [59]. The variation in specific length variants between experimental treatments observed in this work may therefore have functional significance.

Further analysis of the effects of HTT—40 °C on non-mycorrhizal or mycorrhizal grapevines showed a higher number of DEmiRNAs in mycorrhizal (28) than in non-mycorrhizal plants (17), suggesting that mycorrhizal colonization may result in enhanced gene regulation in response to heat stress. Most of the differentially expressed miRNA target genes in mycorrhizal grapevines are transcription factors, genes involved in lipid metabolism and in phosphate starvation response, and genes related to defence mechanisms [60,61]. This indicates that miRNAs extensively regulate plant gene expression during mycorrhizal symbiosis, and that this modulation is dynamic and responsive to environmental cues. In fact, miRNAs’ presence in the phloem suggests that they can act as AMF-triggered long-range signaling molecules [23].

Our results further showed that HTT—40 °C had a greater impact on miRNA expression than mycorrhizal inoculation. This is not surprising, as heat stress has major implications on grapevine growth and berry development, photosynthesis, reproductive development, and oxidative stress [35,36]. Heat stress has also been shown to affect Pi uptake and Pi-related genes and miRNAs, including miR399 [62]. Furthermore, phosphate transporters have been found to be modulated by mycorrhizal colonization under abiotic stress [4,63]. Our prediction that members of the miR399 family target phosphate transporters and that their expression is affected by temperature and mycorrhizal colonization might indicate miR399 involvement in the improved physiological parameters observed in the inoculated plants.

As expected, a considerable number of heat-responsive (up- or downregulated) miRNAs in grapevine leaves were related to the abovementioned developmental or stress related processes. A subset of miRNAs was upregulated by HTT—40 °C in both mycorrhizal and non-mycorrhizal plants, including miR162-3p.1, miR166b, miR167a and miR167a.1, and miR393b-5p. As observed in other species [64], miR166b was predicted to target genes encoding several homeobox-leucine zipper proteins (Table S9), which have significant roles in vascular development, meristem maintenance and mediation of the action of hormones such as auxins [65]. Moreover, genes from this family have been shown to play a role in enhancing the response of plants in warm and dry conditions [66,67]. Auxin regulation in heat-stressed grapevines might also be influenced by the upregulation of miR393 members, which target the auxin receptors TIR1/AFB2. In species such as wheat, miR393 was reported to be repressed by heat stress [68], whereas in switchgrass (*Panicum virgatum*) it was upregulated. It was also observed that miR3633a-5p, which targets gibberellin 2-beta-dioxygenase, was downregulated by temperature. These enzymes play an important role in the gibberellin catabolic pathway, yielding inactive gibberellin products, and therefore may have an effect on plant growth and development [69]. Hence, a fine-tuning regulation of stress-related miRNAs amongst plant species is expected, especially if factors such as tissue type or plant age are taken into consideration.

Fifteen miRNAs showed heat-induced expression exclusively in AMF colonized grapevines, suggesting that mycorrhizal colonization directly modulates plant molecular responses to heat. For instance, miR396 family members were only upregulated by HTT—40 °C in mycorrhizal plants, as were miR164a, miR403f, and miR4995. MicroR396, which is known to affect mycorrhization [70], regulates members of the GRF family of transcription factors, which are associated with growth control of multiple tissues and organs in a variety of species [71]. Interestingly, the overexpression of *GRF15* was shown to increase heat tolerance in transgenic poplar [72]. Furthermore, NAC genes, which are targeted by miR164, also seem to be involved in heat stress tolerance and thermomemory, as was shown in *Arabidopsis* [73].

Interesting too, was the downregulation of three members of the miR398 family, identified exclusively in mycorrhizal grapevine plants subjected to HTT—40 °C (miR398c-3p, miR398c-3p.1, and miR398a-3p.2). MicroRNA398 is particularly well known to respond to heat stress, and targets the closely related copper/zinc superoxide dismutases (SOD) *CSD1* and *CSD2*, and copper chaperone for SOD *(CCS*), that play a role in scavenging superoxide radicals, as well as Cox [74]. Previous studies have reported that these genes are rapidly induced in response to heat [28,75]. Furthermore, miR398 also seems to be involved in mycorrhizal colonization [76]. It has been observed that AMF inoculation significantly increases SOD activity in roots and leaves [77,78,79], thereby enhancing the plant antioxidant protective system. Our results suggest that AMF colonization may increase grapevine tolerance to heat by decreasing miR398 activity and therefore, increasing the activity of putative target genes related to SOD.

Amongst the 32 putative novel miRNAs identified in grapevine leaves, six were found to be already deposited in the miRVIT database, further supporting our novel miRNA prediction. Interestingly, the novel Vvi-4, greatly upregulated by HTT—40 °C, was predicted to target a putative disease resistance RPP13-like protein 1. In maize, a putative disease resistance RPP13-like protein 3 was found to be involved in abscisic acid-regulated heat resistance [80]. It is plausible that the RPP13-like protein 1 is also involved in heat-stress regulation in the grapevine. Our results strongly support the need for further studies on the functions of novel miRNAs in grapevine.

### 3.3. Mycorrhizal Species Influence microRNA Expression under Heat Stress

Recently, it was shown that grapevine inoculation with *R. irregulare* could help to sustain plant growth after a prolonged heat shock (five days at 40/35 °C, day/night), compared to inoculation with *F. mosseae* [39]. However, in the present study, grapevine plants inoculated with these two fungi did not show significant differences in their physiological parameters, and both AMF proved to be equally efficient in promoting plant response to the applied heat stress.

Despite the lack of differences between mycorrhizal plants at a physiological level, we found that plants inoculated with *R. irregulare* presented a higher number of DEmiRNAs between temperature treatments (CT—25 °C and HTT—40 °C) than plants inoculated with *F. mosseae*. The over-expression of a higher number of heat stress-inducible miRNAs belonging to the miR156/miR529/miR535 superfamily (miR156d/g and miR535a) in grapevines colonized by *R. irregulare* is of particular interest. This superfamily shows extremely high sequence identity and is well documented in the modulation of plant growth and development [81]. In accordance with other studies [82], grapevine miR156 members were predicted to target several *SPL* genes. The involvement of the miR156-*SPL* module in heat stress tolerance has been shown in *Arabidopsis*: it modulates the expression of heat-inducible genes such as heat-shock proteins [32] and miR156-overexpressing plants exhibited enhanced tolerance to heat stress and heat stress memory.

The grapevine specific miR3640-5p was the only miRNA found to be downregulated by heat stress in plants inoculated with *F. mosseae*, whereas in *Ri* plants, other miRNAs were also repressed, including: miR3632-5p, miR3627-5p, miR3627-3p, miR3634-5p, and miR397a-5p. Interestingly, the induction of grapevine-specific miRNAs miR3624, miR3633, miR3634, miR3636, and miR3640 have also been observed upon cold treatment [83]. This indicates that those grapevine-specific miRNAs may have, in fact, a role in the response to temperature stress. Gene ontology analysis of the targets of downregulated miRNAs further showed an enrichment in terms such as ‘lignin catabolic process’ and ‘hydroquinone: oxygen oxidoreductase activity’, mostly due to the targeting of *laccases* (*LACs*) by miR397a-5p. *Laccases* encode multicopper oxidases, and in plants, several *LACs* participate in lignin synthesis and metabolism [84]. Despite the absence of genes encoding plant cell wall hydrolytic enzymes in AMF genomes [85], it is plausible that increased temperature differentially affected lignin deposition in *F. mosseae* and *R. irregulare*-inoculated plants, as cell wall loosening might facilitate intercellular fungal colonization.

Finally, it is important to discuss the involvement of auxins, a group of plant hormones with key roles in virtually all aspects of plant growth and development, including responses to environmental cues [86]. Auxins act by promoting the degradation of transcriptional regulators (Aux/IAA proteins), which require TIR1/AFB F-box proteins [87]. Heat-associated regulatory networks in Ri and Fm plants revealed distinct clusters of target genes related to auxin, such as genes encoding for auxin receptors F-box 2 and TIR1, SPL, Homeobox-leucine zipper proteins, and NAC and Cox complex. This strongly supports the role of auxins as well as these genes in grapevine heat stress response. Similarly to our results, in *Arabidopsis* it was shown that genes for F-box proteins are regulated by miR393 and miR394, promoting ubiquitination and proteasome degradation [88]. In wheat, overexpression of the F-box protein TaFBA1 was associated with enhanced antioxidant activity and drought stress tolerance [89]. Moreover, the auxin induced miR164 has been shown to target *NAC* and *CUP-SHAPED COTYLEDON 2* and to regulate stress responses [90]. Homeobox-leucine zipper family members are targeted by miR166 and also respond to auxin [65]. In our study, grapevine miRNAs predicted to target Cox complex included miR393 and miR398. In *Arabidopsis*, miR398 is known to target Cox5b-1 (a subunit of Cox) and is highly involved in heat response [74].

Our results indicate the existence of grapevine-specific miRNAs that may add a functional layer in response to temperature stress, thus contributing to a faster acclimation to high temperatures. However, further studies are still necessary to unveil if the identified miRNAs are variety-specific or if they are common to *V. vinifera* species. In addition, we demonstrate that mycorrhizal symbiotic relationships have implications on grapevine miRNA modulation under heat stress. Furthermore, the differential effects of AMF species on heat stress-regulated miRNA expression undoubtedly expand the current knowledge of the molecular mechanisms involved in symbiosis and plant–heat stress interaction.

The present study has therefore not only laid a foundation for further experimental research and computational analyses of the molecular dynamics of plant–AMF–stress interactions but also provides important first steps toward sustainable breeding practices that cater to abiotic stress tolerance. For example, the development of microRNA-based markers from miR164 or miR396 could lead to a faster selection of grapevine clones showing higher heat tolerance/adaptability to high temperatures, which could be very valuable in the current context of global warming.

## 4. Materials and Methods

### 4.1. Plant Material and Growth Conditions

Un-rooted grapevines of the Touriga Nacional variety grafted onto 1103 Paulsen rootstocks were obtained from a VitiOeste nursery (Portugal) and planted in rooting beds filled with sterile perlite. Touriga Nacional is a red grapevine variety highly cultivated in Portugal [91]. It produces small black/blue berries with high sugar content and aromas, which makes it suitable for Porto wine production (www.infovini.com, accessed on the 8 February 2023).

When the first roots appeared, plants were transplanted to 3 L containers filled with a mixture of sterile peat/perlite (2:1 *v*/*v*) whose pH was corrected to 7.5 with Ca_2_(CO)_3._ At transplant, 12 plants were inoculated with *Rhizoglomus irregulare* (Ri) isolate BEG 72 obtained from the Institute of Agrifood Research and Technology-IRTA (Catalonia, Spain). For this Ri treatment, a total of 5 g of inoculum was added directly to the roots (~100 infective propagules per g of carrier material). For the *Funneliformis mosseae* (Fm) treatment, another subset of 12 plants were inoculated with *F. mosseae* isolate BEG 95 purchased from Symbiom Company (Checz Republic) by adding 10 g of inoculum (~55 infective propagules per g of carrier material). Control treatments (AMF-free inocula) for each inoculum source were established by inoculating plants with a filtrated fungi-free suspension from either *R. irregulare* inoculum (CRi treatment) or *F. mosseae* inoculum (CFm treatment). All plants were maintained under greenhouse conditions for eight months. Average temperature in the greenhouse was 26 ± 5 °C and the average humidity was 58 ± 12%. No artificial light was used. Plants were watered daily during the growing season, and every two weeks a half-strength Hoagland solution was applied [92].

### 4.2. Grapevine Exposure to High Temperature Treatment

After leaf sprouting in the second growing year (eight months after plant inoculation), grapevines were placed in a growth chamber for one week at 25/18 °C day/night and 16 h photoperiod for acclimatization. After this period, half of the plants of each inoculation treatment (Ri, Fm, CRi, CFm) were moved to a new chamber with the following high temperature regime: 18 °C during the night, increasing temperature from 18 °C to 40 °C (0.2 °C per min), four hours at 40 °C, and decreasing temperature from 40 °C to 18 °C (0.2 °C per min). The other half of the plants remained in the growth chamber at 25/18 °C day/night for one additional week. This resulted in a complete randomized factorial experiment with two factors—AMF inoculation and temperature. The eight experimental treatments consisted of *R. irregulare* (Ri) or *F. mosseae* (Fm) inoculation, their respective non-inoculated controls (CRi, CFm), under control temperature (CT—25 °C) or exposed to a high-temperature treatment (HTT—40 °C), with three biological replicates each. Both growth chambers had identical light and humidity conditions (700 photon flux density, 70% relative humidity).

To assess each plant’s physiological status after the one-week temperature trial, the following measurements were obtained from three different apical leaves (the 3rd, 4th, and 5th leaf) of all plants: net photosynthetic rate (P_n_), stomatal conductance (g_s_), transpiration rate (E), and reflectance spectra from the visual and near-infrared regions (Vis-NIR) between 300 and 1150 nm wavelengths. Gas exchange parameters (P_n_, g_s_, and E) were measured with an Infrared Gas Analyzer (Licor 6400, LICOR BioSciences, Lincoln, NE, USA) equipped with a 2 × 3 cm^2^ transparent leaf chamber. Leaf reflectance spectra were collected with a UniSpec-SC (Single Channel) Spectral Analysis System (PP Systems Inc., Amesbury, MA, USA) in the 3rd, 4th, and 5th leaf of each plant, in three different points per leaf. The average reflectance was calculated per leaf and per plant. Several indexes were calculated from those spectra according to Peñuelas et al. [42,93]: Water index (WI) (indicative of plant water status), Chlorophyll index (CHLI) (non-destructive method for leaf chlorophyll assessment), Photochemical Reflectance Index (PRI) (related to xanthophyll cycle and to the dissipation of excess radiation, which provides an estimation of photosynthetic performance), and Normalized Difference Vegetation Index (NDVI) (related to green biomass and used for indirect estimates of photosynthetic capacity and net primary production), according to the following formulas:

WI = *R*900/*R*970

CHL = *R*750/*R*700

PRI = *R*531 − *R*570/*R*531 + *R*570

NDVI = *R*900 − *R*680/*R*900 + *R*680,

where *R* is the reflectance measured at a particular wavelength (nm).

Approximately 2 g of roots were collected from each plant and mycorrhizal colonization rates were determined by root staining with 0.05% Trypan Blue in lactic acid [94], and using the gridline intersect method under a 40× optical microscope (Olympus, Shinjuku City, Tokyo, Japan) [95].

Significant differences amongst treatments for colonization rate, physiological parameters, and reflectance indexes were determined by Two-Way ANOVA using temperature and AMF inoculation as main factors, followed by Duncan’s post hoc test, with a significance of *p* < 0.05.

Additionally, young leaf samples were collected, snap-frozen in liquid nitrogen, and kept at −80 °C until RNA extraction.

### 4.3. Small RNA Library Construction and Sequencing

Total RNA was extracted from grapevine leaves of three plants per experimental treatment using Spectrum^TM^ Plant Total RNA Kit (Sigma-Aldrich, St. Louis, MO, USA) following manufacturer’s instructions. RNA integrity was tested by agarose gel electrophoresis. RNA samples were sent to Fasteris company (Switzerland) to perform small RNA sequencing on an Illumina HiSeq 3000/4000 platform with 1 × 50 bp reads and expected data output of 260–300 million reads per lane. The generated raw small RNA library sequences can be found in the SRA database under the accession number PRJNA814871.

### 4.4. Bioinformatic Analysis and miRNA Identification

Raw sequence reads obtained from Fasteris were processed into clean full length reads using the CLC Genomics Workbench 11 (Qiagen, Hilden, Germany). During this procedure, all low-quality reads including 3′ adapter and 5′ adapter contaminants were removed. Adapter sequences (TGGAATTCTCGGGTGCCAAG) were trimmed from the remaining high-quality sequences. Sequences larger than 30 nt and smaller than 15 nt were discarded. Clean reads were mapped against RFam database (14.5) to remove RNAs other than miRNAs (ribosomal and transfer RNAs, small nuclear and small nucleolar RNAs, repeat sequences, and other non-coding RNAs). The remaining reads were aligned with the mature or precursor miRNAs retrieved from miRBase 22.1 (http://www.mirbase.org, accessed on the 8 February 2023) database, to identify known miRNAs with a maximum of two nucleotide mismatches. The option “Create grouped sample, grouping by Mature” was utilized to merge all length variants (isomiRs) with the same mature sequence to create one expression value for all. The obtained miRNAs were further mapped against *V. vinifera* genome assembly (PN40024.v4) to find the miRNAs’ loci.

### 4.5. Prediction of Novel miRNA

The unclassified tags were processed for novel miRNA prediction. The UEA sRNA toolkit (v4.7) miRCat pipeline was used to predict novel miRNAs, using default plant parameters [96]. Sequences of sRNA were mapped to a *V. vinifera* genome assembly (PN40024.v4). Stringent criteria were used to predict novel miRNAs [45]. RNA sequences are most likely to represent a miRNA when Minimum Fold Energy (MFEI) is more than 0.85 [97]. Moreover, the predicted novel miRNAs were searched in the miRVIT database [46] to annotate novel miRNAs that have been previously reported in grapevine.

### 4.6. Target Prediction of Conserved and Novel miRNAs

psRNATarget software [98] was used to identify potential relationships between conserved and novel miRNAs and their target genes. The *V. vinifera* transcript library (145_Genoscope.12X) was used to identify target genes. To predict target functions, a Blastx with an e-value less than 1E-3 was performed on the NR database. Mapping and Gene Ontology (GO) annotations were added using OmicsBox v2.0.36. software (BioBam Bioinformatics, Valencia, Spain). Kyoto Encyclopedia of Genes and Genomes (KEGG) (https://www.genome.jp/kegg/, accessed on the 8 February 2023) analysis was further used to investigate the metabolic pathways in which the target genes are involved. Predicted target genes for conserved miRNAs were translated into protein to query the Search Tool for the Retrieval of Interacting Genes/Proteins (STRING version 11.5) database [99]. MicroRNAs and interactome data were associated by orthologous proteins matching grapevine with identity > 90%. Stronger protein associations had an edge confidence of 0.900.

### 4.7. Differential Expression Analysis of miRNAs

For a comparative analysis of conserved and novel miRNAs between treatments, the EDGE test implemented on CLC Genomics Workbench 11 and incorporated in the EdgeR package (Robinson et al. 2010) was used. MiRNAs were normalized using reads per million to reduce potential errors before calculating the fold change and *p*-value. A False Discovery Rate (FDR) less than 0.05 was used to identify differentially expressed miRNAs (DEmiRNAs). A General Linear Model analysis (GLM) (Likelihood ratio test) was performed to further understand the interaction of temperature and AMF species on miRNAs’ expression.

### 4.8. Validation of miRNA and Target Gene Expression by RT-qPCR

The abundance of four randomly selected miRNAs (miR164a, miR3630-3p, and miR3640-5p and miR396a-5p) were analysed using pre-designed TaqMan^®^ MicroRNA assays (ThermoFisher Scientific, Waltham, MA, USA), according to the manufacturer’s instructions. The TaqMan^®^ MicroRNA Reverse Transcription Kit was used with each specific reverse PCR primer of each TaqMan^®^ assay. The TaqMan^®^ Universal PCR Master Mix II, No UNG was used with the TaqMan^®^ MicroRNA assays. For the RT-qPCR analysis, three independent biological samples from different plants were used for each treatment. Furthermore, each reaction was carried in triplicate, allowing for experimental replicates. MiR168a-5p and miR162-3p were used as reference for normalisation of expression.

The expression levels of predicted target genes of miR164a, miR3630-3p, miR3640-5p, and miR396a-5p were analysed by RT-qPCR, using SYBRGreen chemistry, as described elsewhere [100]. The genes *Actin* and *Elongation factor 1-alpha* (*Ef1-α*) were used as reference genes. The specificity of the primers was evaluated by melting curve analysis. Primer sequences, amplicon sizes and qPCR amplification efficiencies are shown in Supplementary Table S1. Evaluation of expression stability for the reference miRNAs and genes was done using the statistical application *geNorm* [101].

## Figures and Tables

**Table 1:** *p*-Values of the two-way ANOVA test for the effects of the main factors and their interaction in grapevine physiological parameters and reflectance indexes.

Factor	P_n_	g_s_	E	WI	CHLI	NDVI	PRI
AMF inoculation	0.015 *	0.279	0.320	0.777	0.032 *	0.485	0.222
Temperature	0.269	0.274	0.001 *	<0.001 *	0.133	0.036 *	<0.001 *
Interaction	0.990	0.713	0.658	0.699	0.347	0.715	0.707

AMF: arbuscular mycorrhizal fungi; P_n_: net photosynthesis rate; g_s_: stomatal conductance; E: transpiration rate; WI: water index; CHLI: chlorophyll index; NDVI: normalized difference vegetation index; PRI: photochemical reflectance index. The asterisk indicates a significant effect at *p* < 0.05.

## Data Availability

Data are contained within the article.

## Supplementary Materials

The following supporting information can be downloaded at: https://www.mdpi.com/article/10.3390/plants12050982/s1, Figure S1: Grapevine physiological parameters and reflectance indexes. (A) net photosynthetic rate, (B) stomatal conductance, (C) transpiration rate, (D) water index, (E) chlorophyll index, (F) photochemical reflectance index, and (G) normalized difference vegetation index. Different letters (a, b, c) indicate significant differences amongst treatments, according to Duncan’s post hoc test (*p* < 0.05).; Figure S2: Complete set of MiRNAs identified in the 24 grapevine libraries, showing A) Length distribution of mature miRNAs and B) percentage of first nucleotide bias in the mature miRNAs.; Figure S3: Expression percentage of specific length variants (18–24 nts) for miR157, miR159, miR166, miR167, miR393, miR396, miR399, and miR3627 in the different treatments.; Figure S4: UpSet plot illustrating conserved miRNAs identified in the different grapevine treatments. The bottom left horizontal bars show the total number of miRNAs per treatment. The circles in the panel’s matrix represent the unique and common miRNAs as identified by Venn diagram (unique or overlapping miRNAs). Connected black circles indicate intersection of miRNAs between treatments, while grey circles show no intersection The top vertical columns in each panel summarize the number of miRNAs for each unique or overlapping combination.; Figure S5: Gene Ontology analysis for Biological Process, Molecular Function, and Cellular Component classes for miR3640-5p.; Table S1: Primers used for validation of grapevine miRNA target genes by RT-qPCR.; Table S2: Sequencing and processing summary of the 24 grapevine small RNA libraries.; Table S3: Grapevine microRNAs counts in the 24 leaf samples. miRNAs are grouped by family.; Table S4: Grapevine microRNA sequence variants (isomiRs) detected by small RNA-seq.; Table S5: Predicted novel grapevine miRNAs.; Table S6: Differentially expressed miRNAs between HTT—40 °C and control temperature in non-mycorrhizal plants.; Table S7: Differentially expressed miRNAs between HTT—40 °C and control temperature in mycorrhizal plants.; Table S8: Differentially expressed miRNAs in *R. irregulare* and *F. mosseae* inoculated plants, using temperature as primary experimental factor and mycorrhizal type as secondary experimental factor.; Table S9: Target prediction of grapevine miRNAs.

## References

[1] Schüβler A., Schwarzott D., Walker C. (2001). A New Fungal Phylum, the Glomeromycota: Phylogeny and Evolution* *Dedicated to Manfred Kluge (Technische Universität Darmstadt) on the Occasion of His Retirement. Mycol. Res..

[2] Smith S.E., Read D., Smith S.E., Read D. (2008). 4-Growth and Carbon Economy of Arbuscular Mycorrhizal Symbionts. Mycorrhizal Symbiosis.

[3] Smith S.E., Read D., Smith S.E., Read D. (2008). 5-Mineral Nutrition, Toxic Element Accumulation and Water Relations of Arbuscular Mycorrhizal Plants. Mycorrhizal Symbiosis.

[4] Campos C., Nobre T., Goss M.J., Faria J., Barrulas P., Carvalho M. (2019). Transcriptome Analysis of Wheat Roots Reveals a Differential Regulation of Stress Responses Related to Arbuscular Mycorrhizal Fungi and Soil Disturbance. Biology.

[5] Mathur S., Tomar R.S., Jajoo A. (2019). Arbuscular Mycorrhizal Fungi (AMF) Protects Photosynthetic Apparatus of Wheat under Drought Stress. Photosynth. Res..

[6] Maya M.A., Matsubara Y. (2013). Influence of Arbuscular Mycorrhiza on the Growth and Antioxidative Activity in Cyclamen under Heat Stress. Mycorrhiza.

[7] Bowles T.M., Barrios-Masias F.H., Carlisle E.A., Cavagnaro T.R., Jackson L.E. (2016). Effects of Arbuscular Mycorrhizae on Tomato Yield, Nutrient Uptake, Water Relations, and Soil Carbon Dynamics under Deficit Irrigation in Field Conditions. Sci. Total Environ..

[8] Augé R.M., Toler H.D., Saxton A.M. (2014). Arbuscular Mycorrhizal Symbiosis and Osmotic Adjustment in Response to NaCl Stress: A Meta-Analysis. Front. Plant Sci..

[9] Daynes C.N., Field D.J., Saleeba J.A., Cole M.A., McGee P.A. (2013). Development and Stabilisation of Soil Structure via Interactions between Organic Matter, Arbuscular Mycorrhizal Fungi and Plant Roots. Soil Biol. Biochem..

[10] Nuccio E.E., Hodge A., Pett-Ridge J., Herman D.J., Weber P.K., Firestone M.K. (2013). An Arbuscular Mycorrhizal Fungus Significantly Modifies the Soil Bacterial Community and Nitrogen Cycling during Litter Decomposition. Environ. Microbiol..

[11] Schmitz A.M., Harrison M.J. (2014). Signaling Events during Initiation of Arbuscular Mycorrhizal Symbiosis. J. Integr. Plant Biol..

[12] Vangelisti A., Natali L., Bernardi R., Sbrana C., Turrini A., Hassani-Pak K., Hughes D., Cavallini A., Giovannetti M., Giordani T. (2018). Transcriptome Changes Induced by Arbuscular Mycorrhizal Fungi in Sunflower (*Helianthus annuus* L.) Roots. Sci. Rep..

[13] Schweiger R., Baier M.C., Persicke M., Müller C. (2014). High Specificity in Plant Leaf Metabolic Responses to Arbuscular Mycorrhiza. Nat. Commun..

[14] Salvioli A., Zouari I., Chalot M., Bonfante P. (2012). The Arbuscular Mycorrhizal Status Has an Impact on the Transcriptome Profile and Amino Acid Composition of Tomato Fruit. BMC Plant Biol..

[15] Zouari I., Salvioli A., Chialva M., Novero M., Miozzi L., Tenore G.C., Bagnaresi P., Bonfante P. (2014). From Root to Fruit: RNA-Seq Analysis Shows That Arbuscular Mycorrhizal Symbiosis May Affect Tomato Fruit Metabolism. BMC Genom..

[16] Fiorilli V., Vannini C., Ortolani F., Garcia-Seco D., Chiapello M., Novero M., Domingo G., Terzi V., Morcia C., Bagnaresi P. (2018). Omics Approaches Revealed How Arbuscular Mycorrhizal Symbiosis Enhances Yield and Resistance to Leaf Pathogen in Wheat. Sci. Rep..

[17] Borges F., Martienssen R.A. (2015). The Expanding World of Small RNAs in Plants. Nat. Rev. Mol. Cell Biol..

[18] Zhang B., Pan X., Cannon C.H., Cobb G.P., Anderson T.A. (2006). Conservation and Divergence of Plant MicroRNA Genes. Plant J..

[19] Spanudakis E., Jackson S. (2014). The Role of MicroRNAs in the Control of Flowering Time. J. Exp. Bot..

[20] Lan Y., Su N., Shen Y., Zhang R., Wu F., Cheng Z., Wang J., Zhang X., Guo X., Lei C. (2012). Identification of Novel MiRNAs and MiRNA Expression Profiling during Grain Development in Indica Rice. BMC Genom..

[21] Wang J., Meng X., Dobrovolskaya O.B., Orlov Y.L., Chen M. (2017). Non-Coding RNAs and Their Roles in Stress Response in Plants. Genom. Proteom. Bioinform..

[22] Pagano L., Rossi R., Paesano L., Marmiroli N., Marmiroli M. (2021). MiRNA Regulation and Stress Adaptation in Plants. Environ. Exp. Bot..

[23] Devers E.A., Branscheid A., May P., Krajinski F. (2011). Stars and Symbiosis: MicroRNA- and MicroRNA*-Mediated Transcript Cleavage Involved in Arbuscular Mycorrhizal Symbiosis. Plant Physiol..

[24] Lauressergues D., Delaux P.-M., Formey D., Lelandais-Brière C., Fort S., Cottaz S., Bécard G., Niebel A., Roux C., Combier J.-P. (2012). The MicroRNA MiR171h Modulates Arbuscular Mycorrhizal Colonization of Medicago Truncatula by Targeting NSP2: MiR171h Regulates Fungal Colonization. Plant J..

[25] Mewalal R., Yin H., Hu R., Jawdy S., Vion P., Tuskan G.A., Le Tacon F., Labbé J.L., Yang X. (2019). Identification of Populus Small RNAs Responsive to Mutualistic Interactions With Mycorrhizal Fungi, Laccaria Bicolor and Rhizophagus Irregularis. Front. Microbiol..

[26] Wu P., Wu Y., Liu C.-C., Liu L.-W., Ma F.-F., Wu X.-Y., Wu M., Hang Y.-Y., Chen J.-Q., Shao Z.-Q. (2016). Identification of Arbuscular Mycorrhiza (AM)-Responsive MicroRNAs in Tomato. Front. Plant Sci..

[27] Formey D., Sallet E., Lelandais-Brière C., Ben C., Bustos-Sanmamed P., Niebel A., Frugier F., Combier J.P., Debellé F., Hartmann C. (2014). The Small RNA Diversity from Medicago Truncatula Roots under Biotic Interactions Evidences the Environmental Plasticity of the MiRNAome. Genome Biol..

[28] Guan Q., Lu X., Zeng H., Zhang Y., Zhu J. (2013). Heat Stress Induction of MiR398 Triggers a Regulatory Loop That Is Critical for Thermotolerance in Arabidopsis. Plant J..

[29] Li A.-L., Wen Z., Yang K., Wen X.-P. (2019). Conserved MiR396b-GRF Regulation Is Involved in Abiotic Stress Responses in Pitaya (*Hylocereus polyrhizus*). Int. J. Mol. Sci..

[30] Ravichandran S., Ragupathy R., Edwards T., Domaratzki M., Cloutier S. (2019). MicroRNA-Guided Regulation of Heat Stress Response in Wheat. BMC Genom..

[31] Samad A.F.A., Sajad M., Nazaruddin N., Fauzi I.A., Murad A.M.A., Zainal Z., Ismail I. (2017). MicroRNA and Transcription Factor: Key Players in Plant Regulatory Network. Front. Plant Sci..

[32] Stief A., Altmann S., Hoffmann K., Pant B.D., Scheible W.-R., Bäurle I. (2014). Arabidopsis MiR156 Regulates Tolerance to Recurring Environmental Stress through SPL Transcription Factors. Plant Cell.

[33] Hivrale V., Zheng Y., Puli C.O.R., Jagadeeswaran G., Gowdu K., Kakani V.G., Barakat A., Sunkar R. (2016). Characterization of Drought- and Heat-Responsive MicroRNAs in Switchgrass. Plant Sci..

[34] Trouvelot S., Bonneau L., Redecker D., van Tuinen D., Adrian M., Wipf D. (2015). Arbuscular Mycorrhiza Symbiosis in Viticulture: A Review. Agron. Sustain. Dev..

[35] Carvalho L.C., Coito J.L., Colaço S., Sangiogo M., Amâncio S. (2015). Heat Stress in Grapevine: The Pros and Cons of Acclimation. Plant Cell Environ..

[36] Venios X., Korkas E., Nisiotou A., Banilas G. (2020). Grapevine Responses to Heat Stress and Global Warming. Plants.

[37] Nogales A., Rottier E., Campos C., Victorino G., Costa J.M., Coito J.L., Pereira H.S., Viegas W., Lopes C. (2021). The Effects of Field Inoculation of Arbuscular Mycorrhizal Fungi through Rye Donor Plants on Grapevine Performance and Soil Properties. Agric. Ecosyst. Environ..

[38] Torres N., Antolín M.C., Goicoechea N. (2018). Arbuscular Mycorrhizal Symbiosis as a Promising Resource for Improving Berry Quality in Grapevines Under Changing Environments. Front. Plant Sci..

[39] Nogales A., Ribeiro H., Nogales-Bueno J., Hansen L.D., Gonçalves E.F., Coito J.L., Rato A.E., Peixe A., Viegas W., Cardoso H. (2020). Response of Mycorrhizal ’Touriga Nacional‘ Variety Grapevines to High Temperatures Measured by Calorespirometry and Near-Infrared Spectroscopy. Plants.

[40] Campos C., Cardoso H., Nogales A., Svensson J., Lopez-Ráez J.A., Pozo M.J., Nobre T., Schneider C., Arnholdt-Schmitt B. (2015). Intra and Inter-Spore Variability in Rhizophagus Irregularis AOX Gene. PLoS ONE.

[41] Amiri R., Nikbakht A., Rahimmalek M., Hosseini H. (2017). Variation in the Essential Oil Composition, Antioxidant Capacity, and Physiological Characteristics of Pelargonium Graveolens L. Inoculated with Two Species of Mycorrhizal Fungi Under Water Deficit Conditions. J. Plant Growth Regul..

[42] Peñuelas J., Munné-Bosch S., Llusià J., Filella I. (2004). Leaf Reflectance and Photo- and Antioxidant Protection in Field-Grown Summer-Stressed Phillyrea Angustifolia. Optical Signals of Oxidative Stress?. New Phytol..

[43] De Cannière S., Vereecken H., Defourny P., Jonard F. (2022). Remote Sensing of Instantaneous Drought Stress at Canopy Level Using Sun-Induced Chlorophyll Fluorescence and Canopy Reflectance. Remote Sens..

[44] Tavares C.J., Ribeiro Junior W.Q., Ramos M.L.G., Pereira L.F., Casari R.A.d.C.N., Pereira A.F., de Sousa C.A.F., da Silva A.R., Neto S.P.d.S., Mertz-Henning L.M. (2022). Water Stress Alters Morphophysiological, Grain Quality and Vegetation Indices of Soybean Cultivars. Plants.

[45] Axtell M.J., Meyers B.C. (2018). Revisiting Criteria for Plant MicroRNA Annotation in the Era of Big Data. Plant Cell.

[46] Chitarra W., Pagliarani C., Abbà S., Boccacci P., Birello G., Rossi M., Palmano S., Marzachì C., Perrone I., Gambino G. (2018). MiRVIT: A Novel MiRNA Database and Its Application to Uncover Vitis Responses to Flavescence Dorée Infection. Front. Plant Sci..

[47] Pantaleo V., Szittya G., Moxon S., Miozzi L., Moulton V., Dalmay T., Burgyan J. (2010). Identification of Grapevine MicroRNAs and Their Targets Using High-Throughput Sequencing and Degradome Analysis: Grapevine MicroRNAs and Their Targets. Plant J..

[48] Wang P., Yang Y., Shi H., Wang Y., Ren F. (2019). Small RNA and Degradome Deep Sequencing Reveal Respective Roles of Cold-Related MicroRNAs across Chinese Wild Grapevine and Cultivated Grapevine. BMC Genom..

[49] Urban J., Ingwers M.W., McGuire M.A., Teskey R.O. (2017). Increase in Leaf Temperature Opens Stomata and Decouples Net Photosynthesis from Stomatal Conductance in Pinus Taeda and Populus Deltoides x Nigra. J. Exp. Bot..

[50] Duc N.H., Csintalan Z., Posta K. (2018). Arbuscular Mycorrhizal Fungi Mitigate Negative Effects of Combined Drought and Heat Stress on Tomato Plants. Plant Physiol. Biochem..

[51] Liu D., Zhang D., Liu G., Hussain S., Teng Y. (2013). Influence of Heat Stress on Leaf Ultrastructure, Photosynthetic Performance, and Ascorbate Peroxidase Gene Expression of Two Pear Cultivars (Pyrus Pyrifolia). J. Zhejiang Univ. Sci. B.

[52] Feng B., Liu P., Li G., Dong S.T., Wang F.H., Kong L.A., Zhang J.W. (2014). Effect of Heat Stress on the Photosynthetic Characteristics in Flag Leaves at the Grain-Filling Stage of Different Heat-Resistant Winter Wheat Varieties. J. Agron. Crop. Sci..

[53] O’Brien J., Hayder H., Zayed Y., Peng C. (2018). Overview of MicroRNA Biogenesis, Mechanisms of Actions, and Circulation. Front. Endocrinol..

[54] Budak H., Akpinar B.A. (2015). Plant MiRNAs: Biogenesis, Organization and Origins. Funct. Integr. Genom..

[55] Fard E.M., Moradi S., Salekdeh N.N., Bakhshi B., Ghaffari M.R., Zeinalabedini M., Salekdeh G.H. (2020). Plant IsomiRs: Origins, Biogenesis, and Biological Functions. Genomics.

[56] Baev V., Milev I., Naydenov M., Vachev T., Apostolova E., Mehterov N., Gozmanva M., Minkov G., Sablok G., Yahubyan G. (2014). Insight into Small RNA Abundance and Expression in High- and Low-Temperature Stress Response Using Deep Sequencing in Arabidopsis. Plant Physiol. Biochem..

[57] Perdiguero P., Rodrigues A.S., Chaves I., Costa B., Alves A., María N., Vélez M.D., Díaz-Sala C., Cervera M.T., Miguel C.M. (2021). Comprehensive Analysis of the IsomiRome in the Vegetative Organs of the Conifer Pinus Pinaster under Contrasting Water Availability. Plant Cell Environ..

[58] Sablok G., Srivastva A.K., Suprasanna P., Baev V., Ralph P.J. (2015). IsomiRs: Increasing Evidences of IsomiRs Complexity in Plant Stress Functional Biology. Front. Plant Sci..

[59] Balyan S., Joseph S.V., Jain R., Mutum R.D., Raghuvanshi S. (2020). Investigation into the MiRNA/5′ IsomiRNAs Function and Drought-Mediated MiRNA Processing in Rice. Funct. Integr. Genom..

[60] Pandey P., Wang M., Baldwin I.T., Pandey S.P., Groten K. (2018). Complex Regulation of MicroRNAs in Roots of Competitively-Grown Isogenic Nicotiana Attenuata Plants with Different Capacities to Interact with Arbuscular Mycorrhizal Fungi. BMC Genom..

[61] Xu Y., Zhu S., Liu F., Wang W., Wang X., Han G., Cheng B. (2018). Identification of Arbuscular Mycorrhiza Fungi Responsive MicroRNAs and Their Regulatory Network in Maize. Int. J. Mol. Sci..

[62] Pacak A., Barciszewska-Pacak M., Swida-Barteczka A., Kruszka K., Sega P., Milanowska K., Jakobsen I., Jarmolowski A., Szweykowska-Kulinska Z. (2016). Heat Stress Affects Pi-Related Genes Expression and Inorganic Phosphate Deposition/Accumulation in Barley. Front. Plant Sci..

[63] Szentpéteri V., Mayer Z., Posta K. (2022). Mycorrhizal Symbiosis-Induced Abiotic Stress Mitigation through Phosphate Transporters in *Solanum lycopersicum* L.. Plant Growth Regul..

[64] Li X., Xie X., Li J., Cui Y., Hou Y., Zhai L., Wang X., Fu Y., Liu R., Bian S. (2017). Conservation and Diversification of the MiR166 Family in Soybean and Potential Roles of Newly Identified MiR166s. BMC Plant Biol..

[65] Ariel F.D., Manavella P.A., Dezar C.A., Chan R.L. (2007). The True Story of the HD-Zip Family. Trends Plant Sci..

[66] Ribichich K.F., Chiozza M., Ávalos-Britez S., Cabello J.V., Arce A.L., Watson G., Arias C., Portapila M., Trucco F., Otegui M.E. (2020). Successful Field Performance in Warm and Dry Environments of Soybean Expressing the Sunflower Transcription Factor HB4. J. Exp. Bot..

[67] Wang J., Zhuang L., Zhang J., Yu J., Yang Z., Huang B. (2019). Identification and Characterization of Novel Homeodomain Leucine Zipper (HD-Zip) Transcription Factors Associated with Heat Tolerance in Perennial Ryegrass. Environ. Exp. Bot..

[68] Kumar R.R., Pathak H., Sharma S.K., Kala Y.K., Nirjal M.K., Singh G.P., Goswami S., Rai R.D. (2015). Novel and Conserved Heat-Responsive MicroRNAs in Wheat (*Triticum aestivum* L.). Funct. Integr. Genom..

[69] Harberd N.P., King K.E., Carol P., Cowling R.J., Peng J., Richards D.E. (1998). Gibberellin: Inhibitor of an Inhibitor Of...?. BioEssays.

[70] Bazin J., Khan G.A., Combier J.-P., Bustos-Sanmamed P., Debernardi J.M., Rodriguez R., Sorin C., Palatnik J., Hartmann C., Crespi M. (2013). MiR396 Affects Mycorrhization and Root Meristem Activity in the Legume Medicago Truncatula. Plant J..

[71] Liebsch D., Palatnik J.F. (2020). MicroRNA MiR396, GRF Transcription Factors and GIF Co-Regulators: A Conserved Plant Growth Regulatory Module with Potential for Breeding and Biotechnology. Curr. Opin. Plant Biol..

[72] Zhao Y., Xie J., Wang S., Xu W., Chen S., Song X., Lu M., El-Kassaby Y.A., Zhang D. (2021). Synonymous Mutation in Growth Regulating Factor 15 of MiR396a Target Sites Enhances Photosynthetic Efficiency and Heat Tolerance in Poplar. J. Exp. Bot..

[73] Shahnejat-Bushehri S., Mueller-Roeber B., Balazadeh S. (2012). Arabidopsis NAC Transcription Factor JUNGBRUNNEN1 Affects Thermomemory-Associated Genes and Enhances Heat Stress Tolerance in Primed and Unprimed Conditions. Null.

[74] Zhu C., Ding Y., Liu H. (2011). MiR398 and Plant Stress Responses. Physiol. Plant.

[75] Zhou R., Yu X., Ottosen C.-O., Zhang T., Wu Z., Zhao T. (2020). Unique MiRNAs and Their Targets in Tomato Leaf Responding to Combined Drought and Heat Stress. BMC Plant Biol..

[76] Song F., He C., Yan X., Bai F., Pan Z., Deng X., Xiao S. (2018). Small RNA Profiling Reveals Involvement of MicroRNA-Mediated Gene Regulation in Response to Mycorrhizal Symbiosis in Poncirus Trifoliata L. Raf. Tree Genet. Genomes.

[77] Li Z., Wu N., Meng S., Wu F., Liu T. (2020). Arbuscular Mycorrhizal Fungi (AMF) Enhance the Tolerance of Euonymus Maackii Rupr. at a Moderate Level of Salinity. PLoS ONE.

[78] Wu N., Li Z., Wu F., Tang M. (2016). Comparative Photochemistry Activity and Antioxidant Responses in Male and Female Populus Cathayana Cuttings Inoculated with Arbuscular Mycorrhizal Fungi under Salt. Sci. Rep..

[79] Yeasmin R., Bonser S.P., Motoki S., Nishihara E. (2019). Arbuscular Mycorrhiza Influences Growth and Nutrient Uptake of Asparagus (*Asparagus officinalis* L.) under Heat Stress. HortScience.

[80] Yang H., Zhao Y., Chen N., Liu Y., Yang S., Du H., Wang W., Wu J., Tai F., Chen F. (2021). A New Adenylyl Cyclase, Putative Disease-Resistance RPP13-like Protein 3, Participates in Abscisic Acid-Mediated Resistance to Heat Stress in Maize. J. Exp. Bot..

[81] Wang L., Sun S., Jin J., Fu D., Yang X., Weng X., Xu C., Li X., Xiao J., Zhang Q. (2015). Coordinated Regulation of Vegetative and Reproductive Branching in Rice. Proc. Natl. Acad. Sci. USA.

[82] Xu M., Hu T., Zhao J., Park M.-Y., Earley K.W., Wu G., Yang L., Poethig R.S. (2016). Developmental Functions of MiR156-Regulated SQUAMOSA PROMOTER BINDING PROTEIN-LIKE (SPL) Genes in Arabidopsis Thaliana. PLoS Genet..

[83] Sun X., Fan G., Su L., Wang W., Liang Z., Li S., Xin H. (2015). Identification of Cold-Inducible MicroRNAs in Grapevine. Front. Plant Sci..

[84] Liu Q., Luo L., Zheng L. (2018). Lignins: Biosynthesis and Biological Functions in Plants. Int. J. Mol. Sci..

[85] Tisserant E., Malbreil M., Kuo A., Kohler A., Symeonidi A., Balestrini R., Charron P., Duensing N., Frei dit Frey N., Gianinazzi-Pearson V. (2013). Genome of an Arbuscular Mycorrhizal Fungus Provides Insight into the Oldest Plant Symbiosis. Proc. Natl. Acad. Sci. USA.

[86] Sharma E., Sharma R., Borah P., Jain M., Khurana J.P., Pandey G.K. (2015). Emerging Roles of Auxin in Abiotic Stress Responses. Elucidation of Abiotic Stress Signaling in Plants: Functional Genomics Perspectives, Volume 1.

[87] Tromas A., Paque S., Stierlé V., Quettier A.-L., Muller P., Lechner E., Genschik P., Perrot-Rechenmann C. (2013). Auxin-Binding Protein 1 Is a Negative Regulator of the SCFTIR1/AFB Pathway. Nat. Commun..

[88] Jones-Rhoades M.W., Bartel D.P. (2004). Computational Identification of Plant MicroRNAs and Their Targets, Including a Stress-Induced MiRNA. Mol. Cell.

[89] Zhou S.-M., Kong X.-Z., Kang H.-H., Sun X.-D., Wang W. (2015). The Involvement of Wheat F-Box Protein Gene TaFBA1 in the Oxidative Stress Tolerance of Plants. PLoS ONE.

[90] Hernandez Y., Goswami K., Sanan-Mishra N. (2020). Stress Induced Dynamic Adjustment of Conserved MiR164:NAC Module. Plant-Environ. Interact..

[91] Magalhães N. (2015). Tratado de Viticultura: A Videira, A Vinha e o “Terroir”.

[92] Hoagland D.R., Arnon D.I. (1950). The Water-Culture Method for Growing Plants without Soil.

[93] Peñuelas J., Filella I., Gamon J.A. (1995). Assessment of Photosynthetic Radiation-Use Efficiency with Spectral Reflectance. New Phytol..

[94] Phillips J.M., Hayman D.S. (1970). Improved Procedures for Clearing Roots and Staining Parasitic and Vesicular-Arbuscular Mycorrhizal Fungi for Rapid Assessment of Infection. Trans. Br. Mycol. Soc..

[95] Giovannetti M., Mosse B. (1980). AN EVALUATION OF TECHNIQUES FOR MEASURING VESICULAR ARBUSCULAR MYCORRHIZAL INFECTION IN ROOTS. New Phytol..

[96] Stocks M.B., Mohorianu I., Beckers M., Paicu C., Moxon S., Thody J., Dalmay T., Moulton V. (2018). The UEA SRNA Workbench (Version 4.4): A Comprehensive Suite of Tools for Analyzing MiRNAs and SRNAs. Bioinformatics.

[97] Zhang B.H., Pan X.P., Cox S.B., Cobb G.P., Anderson T.A. (2006). Evidence That MiRNAs Are Different from Other RNAs. Cell. Mol. Life Sci..

[98] Dai X., Zhuang Z., Zhao P.X. (2018). PsRNATarget: A Plant Small RNA Target Analysis Server (2017 Release). Nucleic Acids Res..

[99] Szklarczyk D., Morris J.H., Cook H., Kuhn M., Wyder S., Simonovic M., Santos A., Doncheva N.T., Roth A., Bork P. (2017). The STRING Database in 2017: Quality-Controlled Protein-Protein Association Networks, Made Broadly Accessible. Nucleic Acids Res..

[100] Campos C., Carvalho M., Brígido C., Goss M.J., Nobre T. (2018). Symbiosis Specificity of the Preceding Host Plant Can Dominate but Not Obliterate the Association Between Wheat and Its Arbuscular Mycorrhizal Fungal Partners. Front. Microbiol..

[101] Vandesompele J., De Preter K., Pattyn F., Poppe B., Van Roy N., De Paepe A., Speleman F. (2002). Accurate Normalization of Real-Time Quantitative RT-PCR Data by Geometric Averaging of Multiple Internal Control Genes. Genome Biol..

