# Supplementary material for: Dynamic Regulation of Grapevine’s microRNAs in Response to Mycorrhizal Symbiosis and High Temperature
Source: Plants (Basel). 2023 Feb 21;12(5):982. doi: 10.3390/plants12050982 (PMC10005052; doi:10.3390/plants12050982)
Supplement: Supplementary file 1 [file plants-12-00982-s001.zip › Supplemental Figures.pdf]

Supplemental Figures

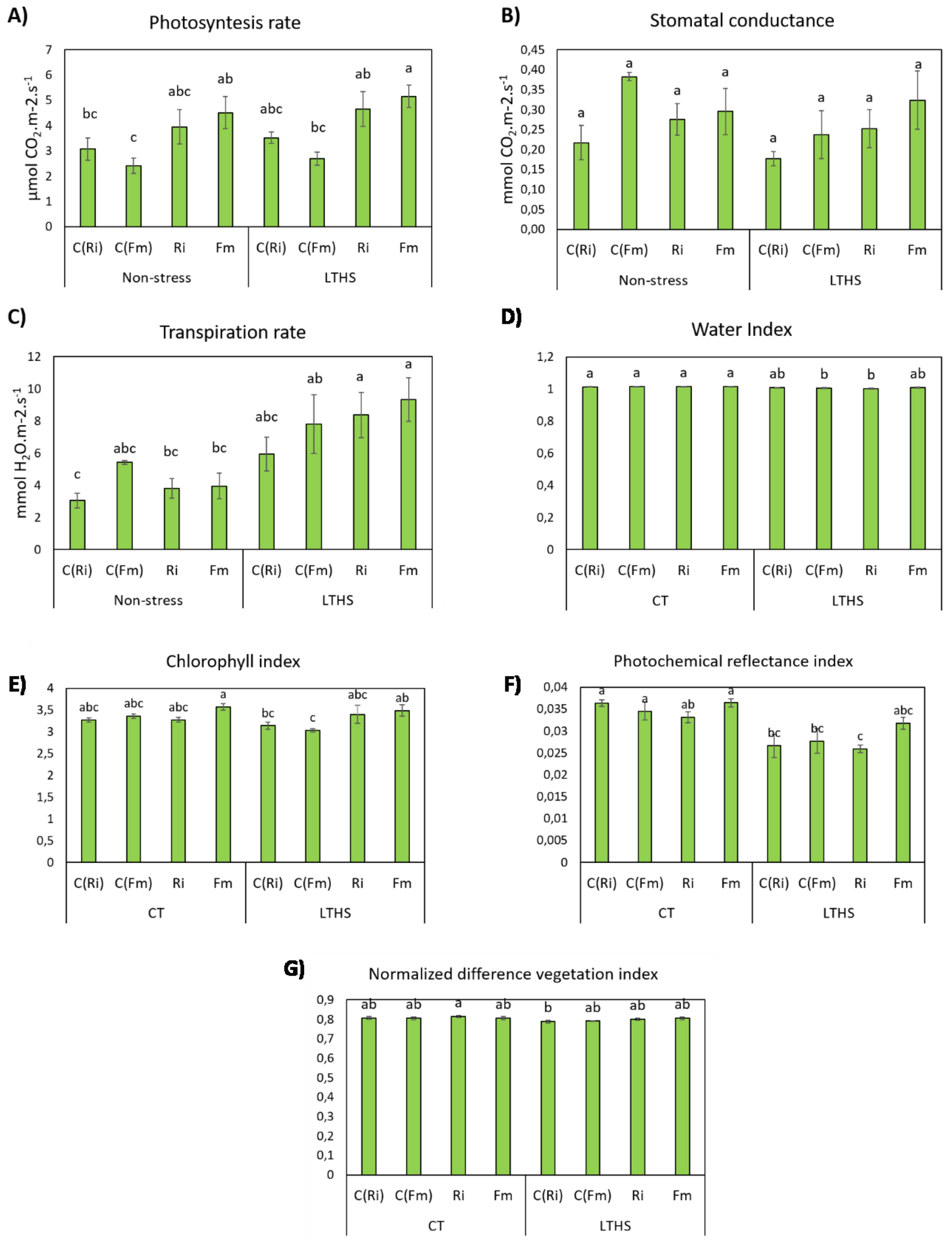

Figure S1. Grapevine physiological parameters and spectra indexes. A) net photosynthetic rate, B) stomatal conductance, C) transpiration rate, D) water index, E) chlorophyll index, F) Photochemical Reflectance Index and G) Normalized Difference Vegetation Index. Different letters (a, b, c) indicate significant differences amongst treatments, as obtained by a Two-Way ANOVA using temperature and mycorrhization as main factors, followed by Duncan's post-hoc test ($P < 0.05$).

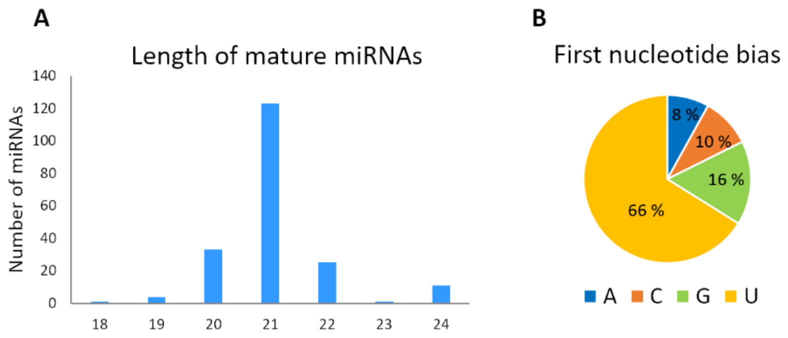

Figure S2. Complete set of MiRNAs identified in the 24 grapevine libraries, showing A) Length distribution of mature miRNAs and B) percentage of first nucleotide bias in the mature miRNAs.

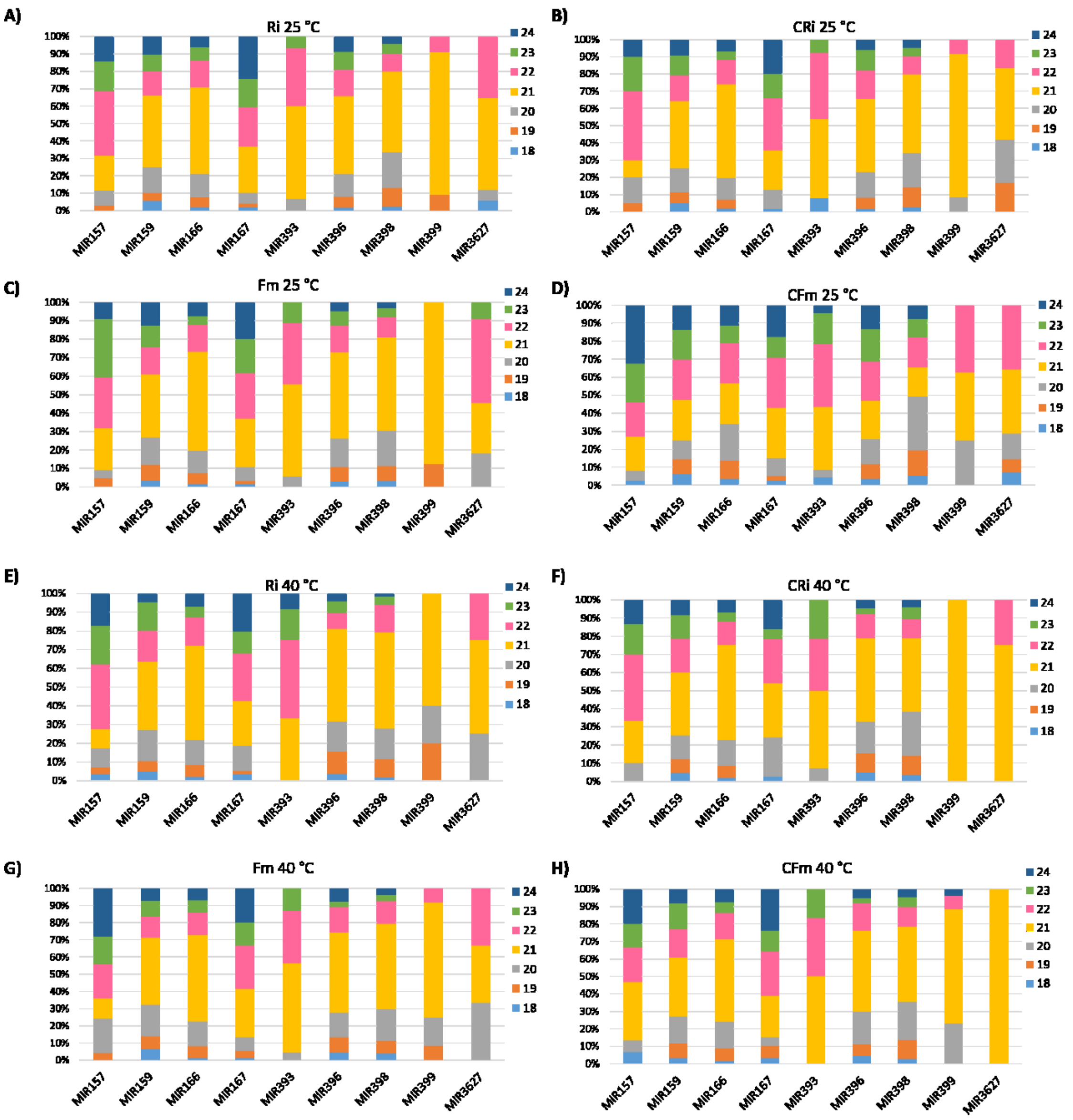

Figure S3. Expression percentage of specific length variants (18 – 24 nts) for miR157, miR159, miR166, miR167, miR393, miR396, miR399 and miR3627 in the different treatments.

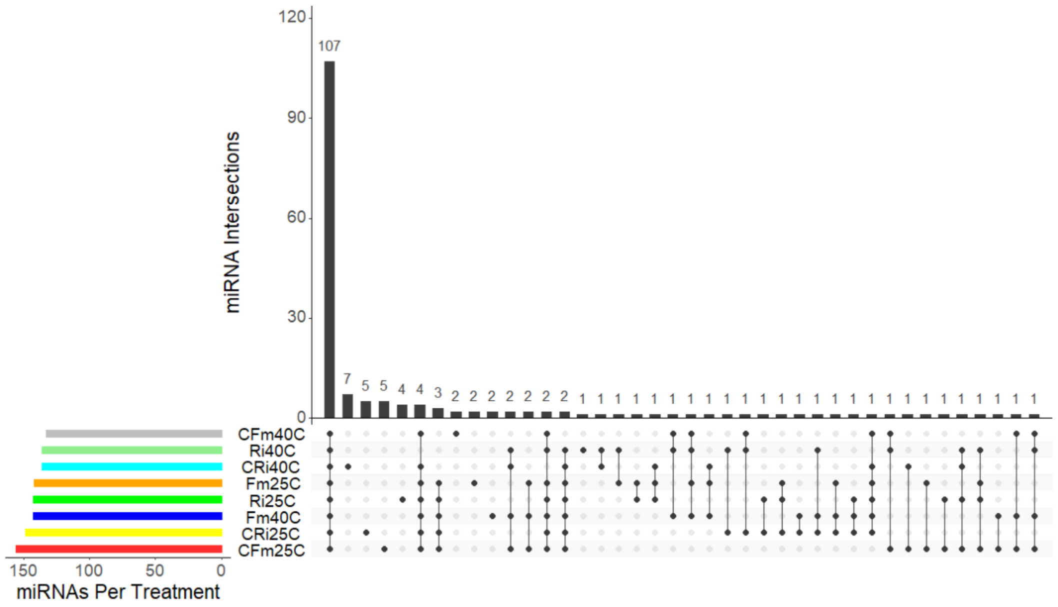

Figure S4. UpSet plot illustrating conserved miRNAs identified in the different grapevine treatments. The bottom left horizontal bars show the total number of miRNAs per treatment. The circles in the panel's matrix represent the unique and common miRNAs as identified by Venn diagram (unique or overlapping miRNAs). Connected black circles indicate the intersection of miRNAs between treatments, while grey circles show no intersection The top vertical columns in each panel summarize the number of miRNAs for each unique or overlapping combination.

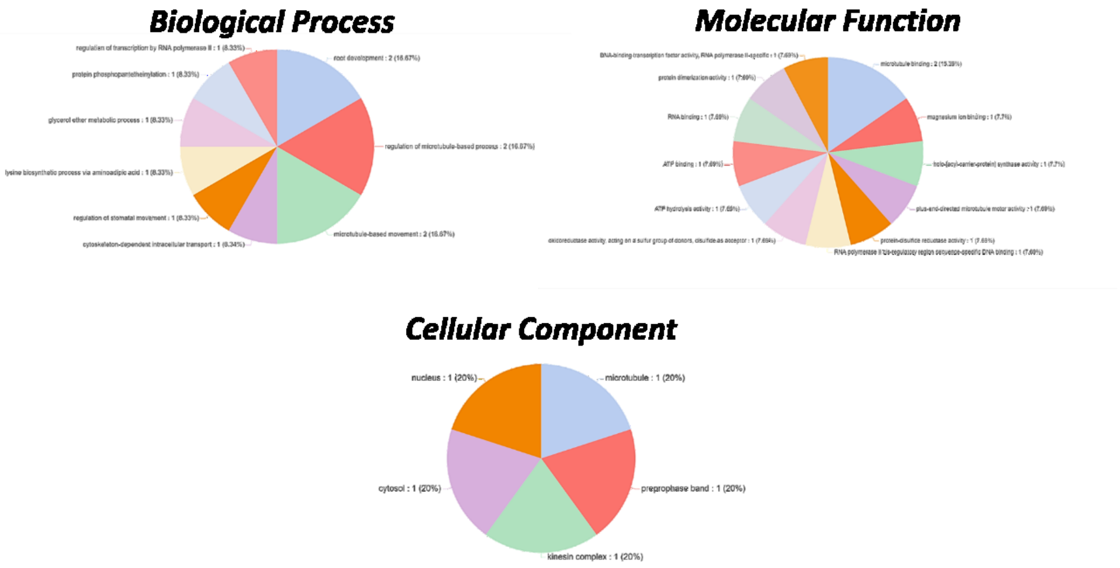

Figure S5. Gene Ontology analysis for Biological Process, Molecular Function and Cellular Component classes for miR3640-5p.
